# Integration of mental health topics in medical postgraduate specialties programms in 50 countries

**DOI:** 10.1192/j.eurpsy.2023.210

**Published:** 2023-07-19

**Authors:** G. Heinze

**Affiliations:** Division de posgrado, Facultad de Medicina UNAM, Mexico-City, Mexico

## Abstract

**Abstract:**

The prolongation of life expectancy has considerably increased the prevalence of chronic diseases and therefore the concomitant presence of these, this usually predicts a more reserved prognosis for both conditions and can lead to greater complications, more complex treatments (4,5) and therefore more expensive and with considerable delays in recovery.(6)

The comorbidity of physical and mental illnesses is common, as both are often interrelated.

One of the ways to ensure handling comprehensive and personalized comorbidity should be detected especially at the first level of care for timely treatment, It is the inclusion of mental health topics in the curricula of doctors in training in a specialty.

**Objective:**

Establish which programs of the medical and surgical specialties include topics related to mental health in their academic training, in order to strengthen the recovery process of patients

**Methods:**

A descriptive study of the academic programs of Medical specialization in 50 countries. The questions we asked ourselves were:

1. What medical specialties contain mental health topics in their training program?

How many medical specialties have included the following mental health topics in their regular graduate program: affective disorders, anxiety disorders, psychosomatic disorders, substance use disorders, violence and palliative care?

What other mental health topics are included in the different medical specialties?

**Discussion:**

The results obtained indicate the importance of mental health in different states of physical health and especially to be taken into account by decision makers in health policies. Comorbidity in current medicine should be taken into consideration more objectively and thus favor the reduction of the suffering of the sick person. We know that an associated mental and physical condition can give atypical presentations that hinder diagnosis, clinical severity, and response to treatment and therefore may lead to increased utilization of health services. However, the fragmentation of medicine into increasingly limited specialties restricts the ability to see the patient holistically and therefore the decrease in the quality of medical care, increase in costs and delay in the recovery process.

**Disclosure of Interest:**

None Declared

